# Plasma image edge detection based on the visible camera in the EAST device

**DOI:** 10.1186/s40064-016-3697-9

**Published:** 2016-12-01

**Authors:** Shuangbao Shu, Chongyang Xu, Meiwen Chen, Zhendong Yang

**Affiliations:** 1 School of Instrument Science and Opto-Electronics Engineering, Hefei University of Technology, Tunxi Road 193, Baohe District, Hefei, 230009 Anhui Province People’s Republic of China; 2 Institute of Plasma Physics, Chinese Academy of Sciences, Shushanhu Road 350, Shushan District, Hefei, 230031 Anhui Province People’s Republic of China; 3 College of Science, Donghua University, North Renmin Road 2999, Songjiang District, Shanghai, 201620 People’s Republic of China

**Keywords:** EAST, Tokamak, Plasma image, Sobel algorithm, Edge detection

## Abstract

The controlling of plasma shape and position are essential to the success of Tokamak discharge. A real-time image acquisition system was designed to obtain plasma radiation image during the discharge processes in the Experimental Advanced Superconducting Tokamak (EAST) device. The hardware structure and software design of this visible camera system are introduced in detail. According to the general structure of EAST and the layout of the observation window, spatial location of the discharging plasma in the image was measured. An improved Sobel edge detection algorithm using iterative threshold was proposed to detect plasma boundary. EAST discharge results show that the proposed method acquired plasma position and boundary with high accuracy, which is of great significance for better plasma control.

## Background

**Fig. 1:**
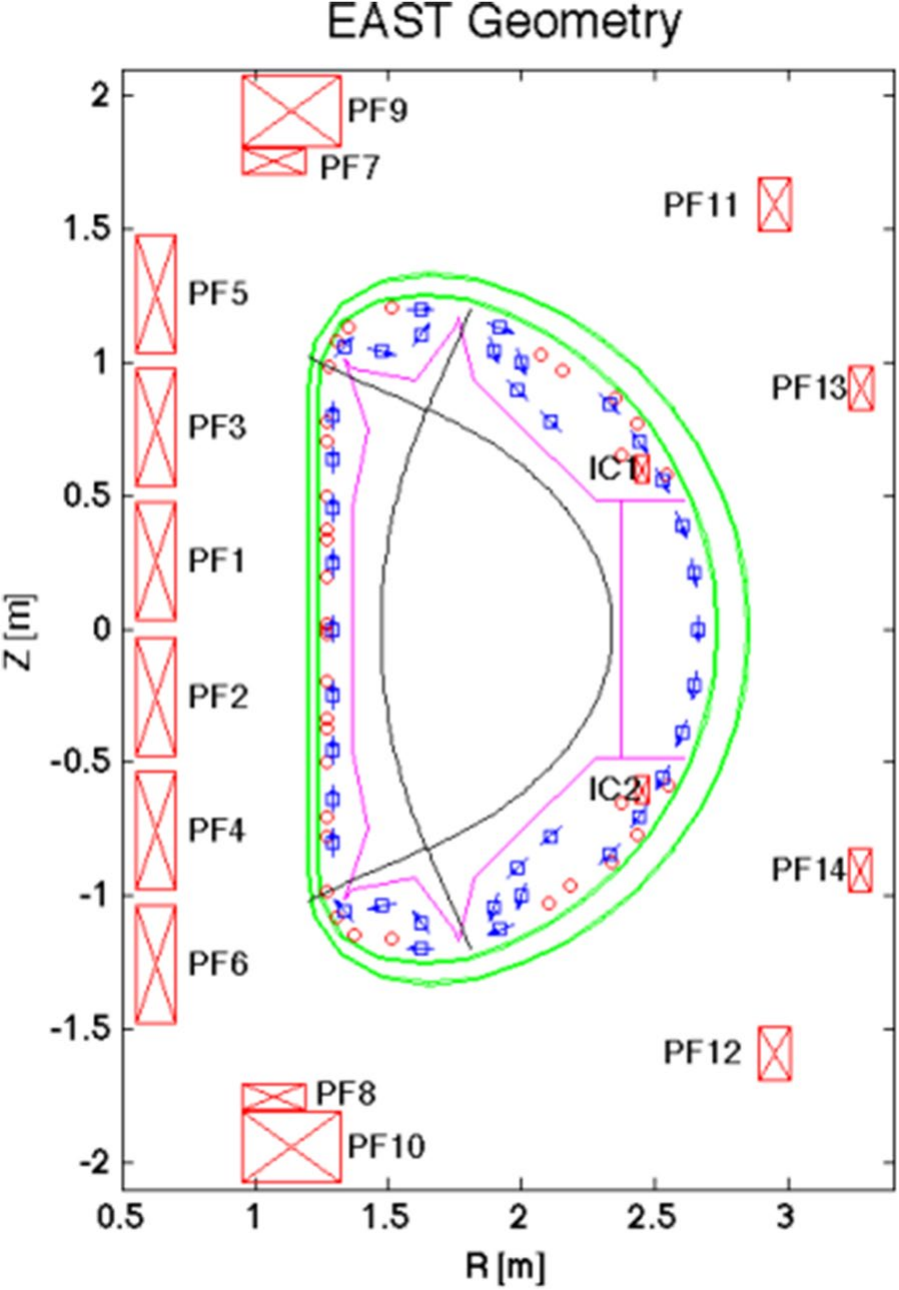
EAST cross section and magnetic diagnostics distribution with flux loops (*circle*) and magnetic probes (*square*)

The Experimental Advanced Superconducting Tokamak (EAST) was built by the Institute of Plasma Physics, Chinese Academy of Sciences, which aims at the demonstration of long pulse stable high-performance plasma operation, and thus provides an important test bed to address key physics and technology issues for next-step fusion devices (Wan 2009; Wan et al. 2013). EAST is the first fully superconducting Tokamak device with advanced divertor configuration and heating scheme similar to the International Thermonuclear Experimental Reactor (ITER). In EAST, 38 poloidally aligned magnetic probes measuring tangential field and 35 flux loops measuring poloidal flux are mounted on the vacuum vessel as shown in Fig. 1. The plasma current and poloidal coil currents are measured by rogowski coils. The EFIT (Equilibrium FITing code) reconstruction provides a least square best fit to the diagnostic data and satisfies the model given by the Grad-Shafranov equation. From such full reconstruction calculation, the plasma pressure, current flux function, internal inductance and the parameters of plasma shape and position can be obtained (Qian et al. 2010; Xiao et al. 2012). However, so heavy computation is not fast enough to plasma control. Thus, the RTEFIT (Real-Time EFIT) algorithm modified from this offline EFIT is used for the fast equilibrium solution in the plasma feedback control. The real-time reconstruction algorithm consists of a fast loop and a slow loop running on two CPUs separately. The fast loop does the fitting calculation for poloidal current source including external coils and plasma current in each grid. And the slow loop completes the steps required in the reconstruction iteration to prepare a new data set including response vector and normalized flux for fast loop. In RTEFIT code, the most important modifications to offline EFIT are the one iteration calculation and the reuse of the data set in fast loop. Each time the fast loop is executed with a new set of diagnostic data, the same data set is reused until a new one is updated by the slow loop. The plasma current and position control generally used is RZIP control. The control parameters are feedback controlled by adjusting the current in poloidal field (PF) coils. The requested PF coil current is composed of feed-forward part and the feedback part. In a control cycle, the plasma current Ip is measured directly from rogowski coil, but the radial and vertical position of plasma current center can be calculated by the estimator using magnetic diagnostic data. With PID (Proportion Integration Differentiation) operation and decoupling calculation, the requested value for PF coil current is achieved. Then the corresponding command for power supply is generated with PF current by PID calculation.

The calculation of EFIT (including offline EFIT and RTEFIT) requires a large number of electromagnetic measurement data from magnetic probes, flux loops, etc. The plasma-forming process is quite complex in the Tokamak start-up phase. The start-up plasma is not in equilibrium state, and the result of magnetic measurement hardly reflects the real situation of plasma current because of the existence of induced eddy current in the vacuum chamber wall (Liu et al. 2008; Zhou et al. 2015). Thus, the calculated result from EFIT is inaccurate and unusable for reliable plasma current and its position control. In addition, during the discharge process, the distance from the outer closed magnetic surface to the inner wall of mid-plane of the high field side (also called as Gap) is a very important parameter. During the preliminary discharge stage, the Gap influences the initiation of plasma, and accurate Gap control becomes helpful for reliable plasma current and position control. EFIT is also one of the methods to get Gap (Qian et al. 2009). Because the Gap will influence the initiation of plasma, it is more meaningful to obtain the accurate distance between plasma and inner wall of the EAST.

A fast visible camera, as a kind of imaging device, can obtain the radiation images in real-time during the plasma discharge (Jia et al. 2015; Yuan et al. 2013; Chapman et al. 2014). This paper mainly discussed how to acquire the plasma image and detect the spatial position of plasma boundary in EAST. A fast image acquisition system for EAST was introduced in this paper. A Sobel edge detection method with improved iterative thresholding algorithm was proposed in real-time plasma boundary detection. According to the EAST device structure, spatial location in the image was calibrated. Gap can also be obtained. It has considerably practical meaning for further plasma control.

The rest of the present paper is organized as follows. According to the structure of the EAST device and the position of the observation window, a fast camera image acquisition system and the camera calibration were presented in second section. In third section, the plasma image edge detection algorithm is described in detail. The experimental results and discussions are given in fourth section. Finally, the conclusion is drawn in fifth section.

## Structure of the EAST visible camera system

### The visible camera set-up

**Fig. 2:**
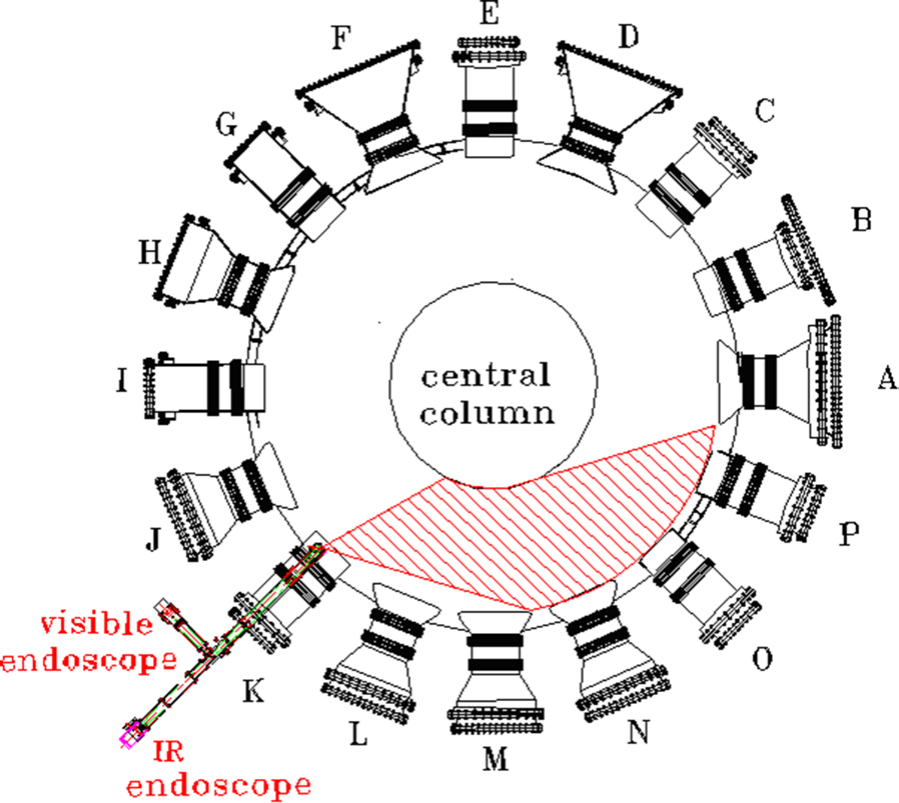
Top view of visible/IR endoscope on EAST

The EAST device can accommodate both single null (SN) and double null (DN) configurations. It was designed for steady-state divertor operation for long pulses of 1000 s and has achieved 411 s in the 2012 experimental campaign. EAST has a major radius R = 1.7–1.9 m and a minor radius a = 0.4–0.45 m, with triangularity δ = 0.4–0.7, elongation κ up to 1.9, maximum plasma current Ip = 1 MA and maximum toroidal field Bt = 3.5 T (Wu 2007). According to the structure of the EAST device and the position of the observation window, the visible and the infrared (IR) cameras share the same window. From the 2014 EAST campaign, this integrated system located in the horizontal K window, can monitor divertors/limiters and antenna ports of the Lower Hybrid Wave (LHW) on the N, O, P three windows. The specific distribution in EAST is shown in Fig. 2. The integrated visible/IR endoscope system consists an integrated endoscope, an infrared camera, and a high speed visible camera. The cameras share only one pinhole to get wide field angle. The wide field of view (47° × 58°) makes it possible to monitor the temperature evolution on the upper divertor targets, lower divertor targets, high field side (HFS), and the limiter simultaneously.

The visible/IR endoscope system including the visible camera, the IR camera and the spectroscope has been installed on the EAST device for the first time in 2014 EAST campaign. The IR camera used in this system is a FLIR SC700BB (2.5–5.0 μm IR ranges). The maximum frame rate is up to 2.9 kHz with a 132 × 3 pixels sub-window and 115 Hz for full-frame (640 × 512 pixels). The visible camera is a Phantom V710, and the maximum frame rate at full resolution (1280 × 800 pixels) is up to 7530 Hz. The spatial resolution of this system along the divertor plate in the poloidal direction is 4 mm for the IR camera and 3 mm for the visible camera. The visible/IR integrated endoscope system can monitor discharge process and the temperature distribution of the first wall and the divertor targets in real-time. In this paper, the authors focus on the observation of the visible camera system.

**Fig. 3:**
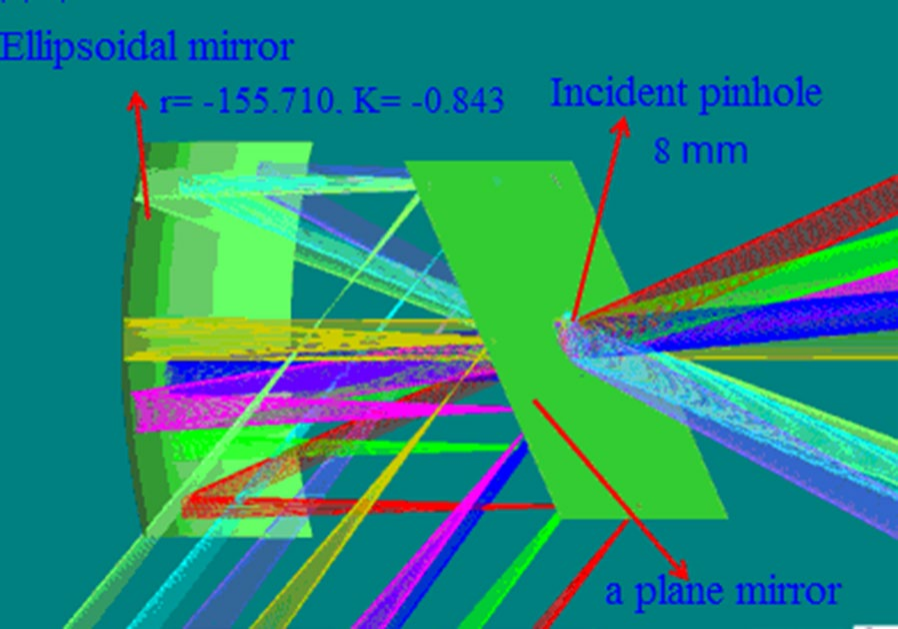
The field of view light source and the front end of the endoscope

As is seen from Fig. 2, the field of view is not parallel to the endoscope. The main part of the optical path is reflective, and the reflection uses small aperture incident. The front end of the endoscope, as shown in Fig. 3, with one window in the infrared and visible, requires only a small opening to observation. The small opening can reduce the neutron radiation, which is also an important advantage in this design. Seen from Fig. 3, the light sources are incident from the pinhole. The diameter of the pinhole designed is 8 mm, which is arranged on the plane mirror. The light passes through the pinhole, and reaches the surface of the ellipsoidal mirror. The light is reflected to the plane mirror and be close parallel to the endoscope. Because the gold film has a strong reflectivity in the infrared wave band, nearly 98%, all the mirrors are coated with gold. Due to the temperature variation of the internal Tokamak, the high temperature wall runs up to 200 °C, and the low temperature wall runs to be less than 60 °C. If the optical materials deformed, it will seriously affect the quality of cameras imaging. So, the glass ceramics with almost zero thermal deformation were selected as the mirror substrate materials. With a comprehensive consideration of the geometric imaging effect and the limited size of the endoscope, diameter of the pinhole is designed to be 8 mm.

### Hardware construction of the visible camera system

**Fig. 4:**
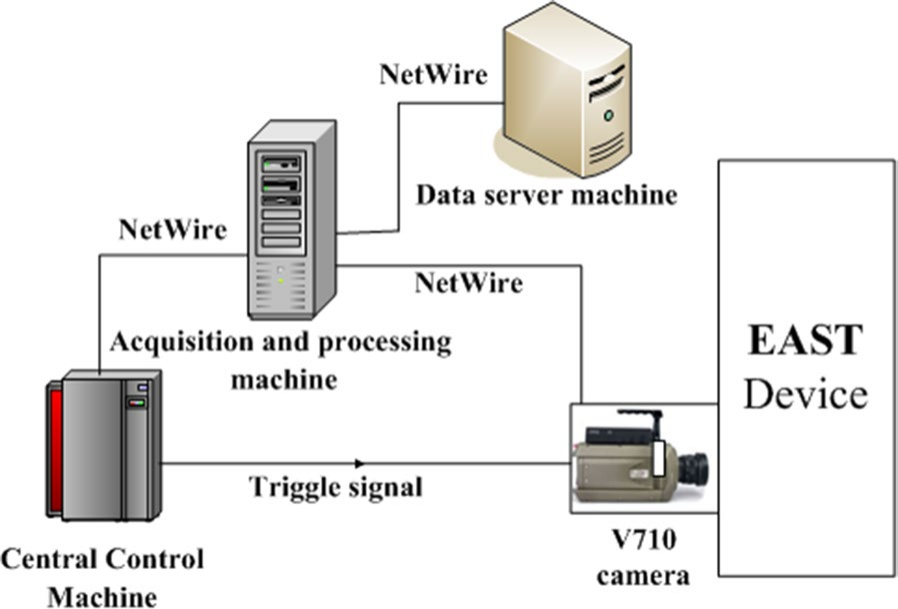
Hardware construction of the visible camera system

In the EAST Tokamak device, many subsystems, such as central timing system, plasma physics diagnosis system, lower hybrid wave system and some other auxiliary systems, are designed as distributed systems. The visible camera system is also designed as a distributed system. The hardware structure of the system mainly includes visible camera, acquisition and processing machine, and data server machine are shown in Fig. 4. The system also needs to process signals from the Central Control Machine (CCM, supplies shot number, trigger signal, synchronous clock, etc.). The Phantom V710 camera is selected as the visible camera, due to its advantages of small size, low weight, fast speed and high imaging quality. The highest sampling frequency can reach to 13 kHz with image 800 × 600 and 50 kHz with image 640 × 208. Captured images can be transmitted through gigabit network with speed up to 100,000 fps. The image sampling and transmission speed can meet the real-time experiment demands.

The CCM is the central control machine in the operation of the EAST experimental device, which is mainly responsible for the unified control and management of all subsystems in the experiment. During the discharge process, the CCM supplies shot number and acquisition time to image acquisition machine and sends trigger signal to the visible camera. The image acquisition machine is responsible for image acquisition, image processing and data uploading to the data server machine.

When an experiment shot starts, the visible camera begins to wait for a trigger from the CCM. Once the trigger arrives, the visible camera begins to capture images and get information about the discharging plasma by real-time image processing. When the shot ends, the camera system immediately sends images and the processing results to the data server machine for further analysis by physical researchers. The data server machine has a huge storage device for storing discharging images and processing the results.

### Software design of the visible camera system

**Fig. 5:**
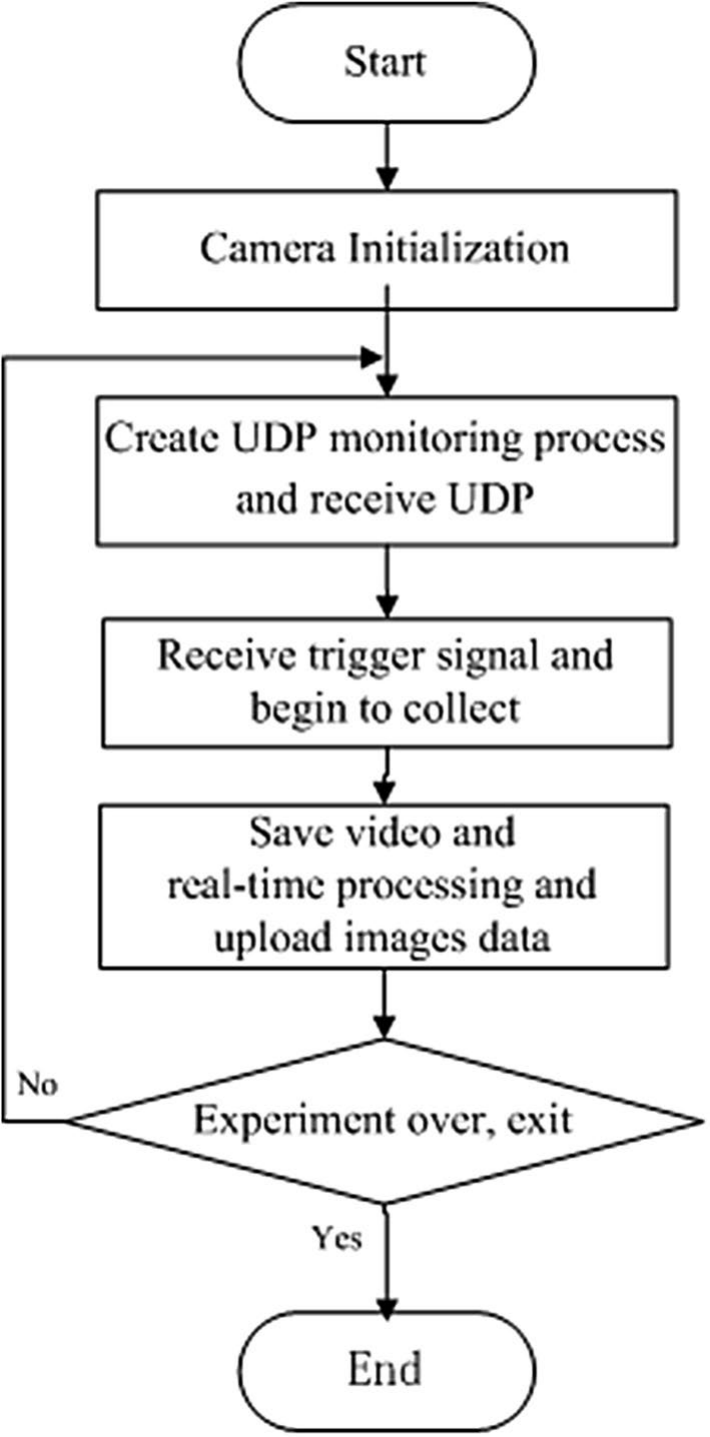
Images acquisition and processing work flow

In order to acquire and process the real-time images, Visual Studio 2008 as development tool and multithreading technology are used in the software program development. The software flowchart is shown in Fig. 5 and detailed descriptions are as follows:

1. V710 camera initialization. Parameters such as sampling rate, exposure time, contrast ratio, are set to proper values.
2. UDP monitoring. UDP monitoring process is created to receive UDP data from CCM, including shot number and acquisition time.
3. Waiting for the trigger from CCM. The V710 camera waits for the start signal before image acquisition. Once the trigger signal arrives, the system starts image acquisition.
4. Video storage and image processing. Discharge video is saved and real-time processing is performed to detect plasma image edge, compress and save images.
5. Uploading image data. When a discharge is finished, the system uploads the image data to the data server machine. If the discharge continues, the system returns to step (2) and repeats UDP monitoring for the next discharge. Otherwise, the system exits.

### The visible camera calibration

**Fig. 6:**
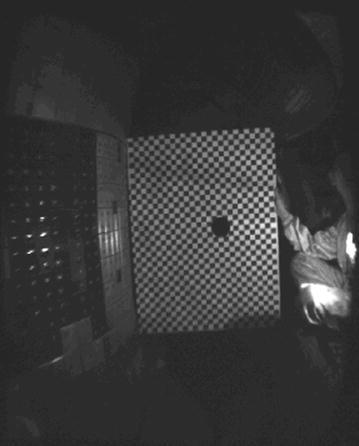
Calibration target location

In order to obtain the internal space position of the EAST device form the images, the camera should be calibrated. We use a calibration board with 2D grid to demarcate the spatial position inside the device. The calibration board is 38 × 28 black and white square grid, and the grid size is 28.0 mm × 28.0 mm. Because the camera is used to detect the radiation of the plasma, the surface of the calibration board should be parallel to the light entrance of the camera and the inner side of the calibration board should be on the tangent line of the inner wall of the high field side. The spatial position of the whole calibration board in vacuum chamber is shown in Fig. 6.

**Fig. 7:**
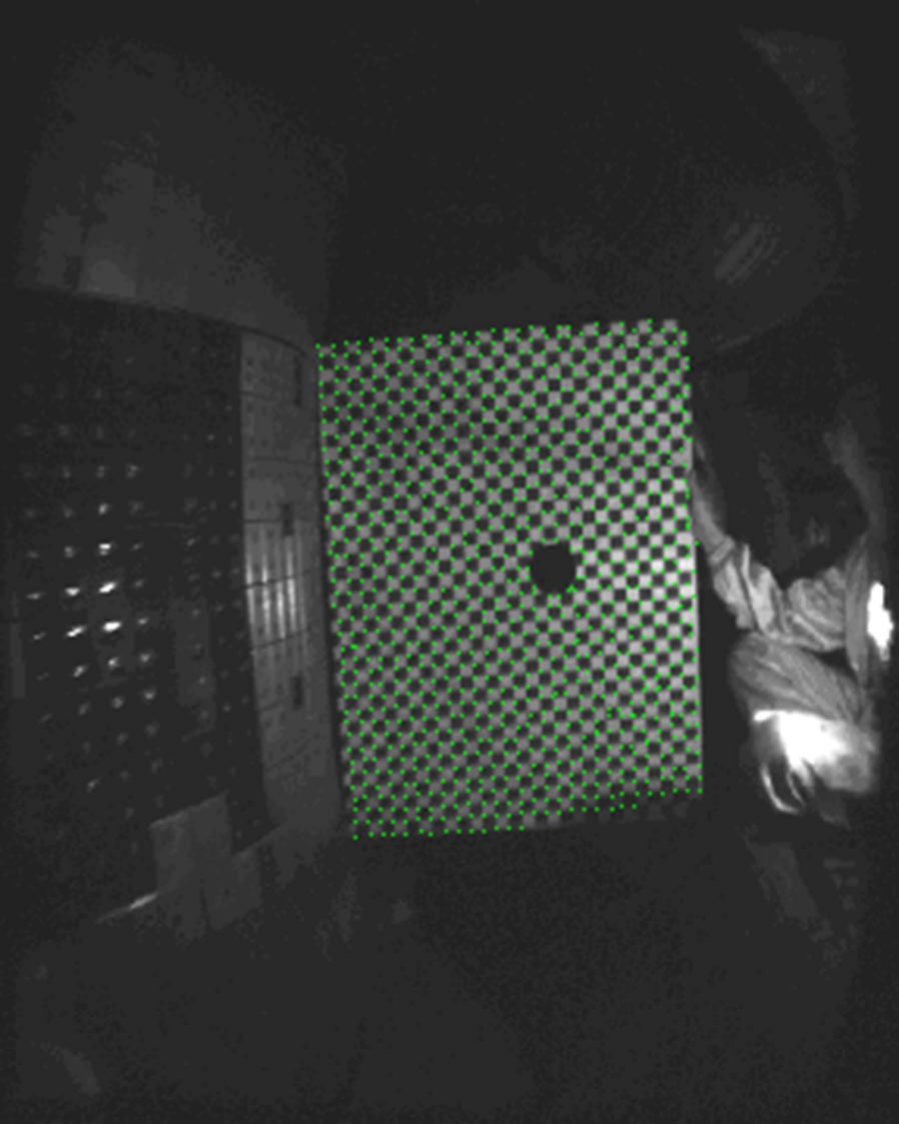
Corner recognition

Through square grid corner detection to the calibration board, the pixel number of a square grid will be obtained in the image. Through contrasting with the actual grid size, each pixel in an image represents certain space size in the device. Figure 7 shows the result of the grid corner recognition. Suppose the number of pixels in a square grid is *n*, the actual size of a pixel is *d* (mm), then

1 $$d^{2} \times n = 28.0 \times 28.0\;\left( {\text{Unit: mm}} \right)$$

The actual size of a single pixel is:

2 $$d = \sqrt {{{28.0^{2} } \mathord{\left/ {\vphantom {{28.0^{2} } n}} \right. \kern-0pt} n}} \;\left( {\text{Unit: mm}} \right)$$

Due to the influence of image quality, the maximum pixel number in a grid is 96, and the minimum is 92. So *d*_max_ = 2.92 mm, and *d*_min_ = 2.86 mm.

The maximum pixel deviation of the actual distance is 0.06 mm. By calculation, the maximum number of pixels between the outer closed magnetic field surface and the inner wall of the high field side of the device is about 40–50, so the maximum error is about 2.40–3.00 mm. Based on all statistical pixel numbers, the average size of a pixel is 2.88 mm.

## Plasma edge detection

As for image edge detection, it is very important to choose a suitable threshold value (Wang et al. 2005; Obrien et al. 1993). This paper applies a modified iterative algorithm to obtain the most reasonable threshold value, which can divide an image into two parts as background and goal. Then a modified Sobel algorithm is applied to complete the plasma edge detection.

### The image threshold segmentation

Before processing threshold segmentation, some pre-processing steps for the original image are required. First, the original image is changed into gray-scale image. Then, noise points made by stray light and camera noise in the image are removed by weighted median filtering method.

After pre-processing, the plasma image is processed by threshold segmentation. Threshold iteration is used for threshold segmentation. The iterative algorithm is equivalent to the mathematical approach. For each image, there is a reasonable threshold value. Suppose the ideal value for threshold value is *T*. First, according to a certain rule, get an initial threshold value *t* for the image and modify *t* until it is close enough to *T*. Assume that the number of pixels with gray value *i* (*i* = 0, …, 255) is *P*_*i*_, *T*_*n*−1_ and *T*_*n*_ are the results of the *n* − 1th and *n*th iterations respectively. Then the optimal threshold value can be expressed as (3) by using the iteration method:

3 $${T_n} = {{\left( {\frac{{\sum\nolimits_{i < {T_{n - 1}}} {{P_i} \times i} }}{{\sum\nolimits_{i < {T_{n - 1}}} {{P_i}} }} + \frac{{\sum\nolimits_{i > {T_{n - 1}}} {{P_i} \times i} }}{{\sum\nolimits_{i > {T_{n - 1}}} {{P_i}} }}} \right)} \mathord{\left/ {\vphantom {{\left( {\frac{{\sum\nolimits_{i < {T_{n - 1}}} {{P_i} \times i} }}{{\sum\nolimits_{i < {T_{n - 1}}} {{P_i}} }} + \frac{{\sum\nolimits_{i > {T_{n - 1}}} {{P_i} \times i} }}{{\sum\nolimits_{i > {T_{n - 1}}} {{P_i}} }}} \right)} 2}} \right. \kern-\nulldelimiterspace} 2}$$

If the histogram of the image has two obvious peaks, we can quickly get the satisfactory result. But the original images probably have various disturbances, such as shadow, uneven luminance, and different contrast ratio. If a fixed threshold is selected for image segmentation, the result may not be acceptable for all images. So we proposed an improved threshold algorithm, which can select different threshold based on image features during plasma discharge.

The first step is to calculate the average threshold value for segmentation in the target area, and then obtain the best threshold value using iterative algorithm. The main part of the algorithm is described as follows:

1. Calculate the maximum gray value *P*_max_ and minimum gray value *P*_min_, and set the initial threshold value *t*_0_ = (*P*_max_ + *P*_min_)/2. According to *t*_0_, segment the general position during the plasma discharge and regard it as the target image.
2. According to threshold value *t*_0_, segment the target image into foreground and background, and then calculate the average gray value *E*_0_ and *E*_*b*_.
3. Calculate the new threshold value *t*_1_ = (*E*_0_ + *E*_*b*_)/2.
4. If *t*_1_ = *t*_0_, the calculated value is the best threshold value. Otherwise, go back to step (2), change *t*_0_ to *t*_1_ and continue the iteration until *t*_*k*+1_ = *t*_*k*_.
5. Use the best threshold value *t*_*k*_ or *t*_*k*+1_to make the image binarization and then output the processed image.

### Edge detection using the improved Sobel algorithm

After threshold segmentation of the image, the improved Sobel algorithm is used to detection the plasma boundary. The principle of Sobel algorithm is as follows:

Sobel operator is the first-order derivation of the edge detection operator, which contains two groups of 3 × 3 matrixes with the horizontal and vertical matrix respectively. Sobel operator and the image convolution can get the horizontal and vertical brightness difference approximation respectively. Assume that the original image is represented by A, *G*_*x*_ and *G*_*y*_ represent the gray values for horizontal and vertical edge detection, then we can get:

4 $$G_{x} = \left[ {\begin{array}{*{20}c} { - 1} &;\quad 0 &;\quad 1 \\ { - 2} &;\quad 0 &;\quad 2 \\ { - 1} &;\quad 0 &;\quad 1 \\ \end{array} } \right] \times A,\quad G_{y} = \left[ {\begin{array}{*{20}c} 1 &;\quad 2 &;\quad 1 \\ 0 &;\quad 0 &;\quad 0 \\ { - 1} &;\quad { - 2} &;\quad { - 1} \\ \end{array} } \right] \times A$$

Combine the horizontal and vertical gray values of each pixel with formula (5) to calculate the gray value of the pixel:

5 $$G = \sqrt {G_{x}^{2} + G_{y}^{2} }$$

Usually, in order to improve computational efficiency, the approximate value is used:

6 $$\left| G \right| = \left| {G_{x} } \right| + \left| {G_{y} } \right|$$

If G is greater than a threshold gradient, this point (x, y) is regarded as the edge point. Then the Formula (7) is used to calculate the gradient direction:

7 $$\theta = \arctan \left( {\frac{{G_{y} }}{{G_{x} }}} \right)$$

## Experimental results and discussion

**Fig. 8:**
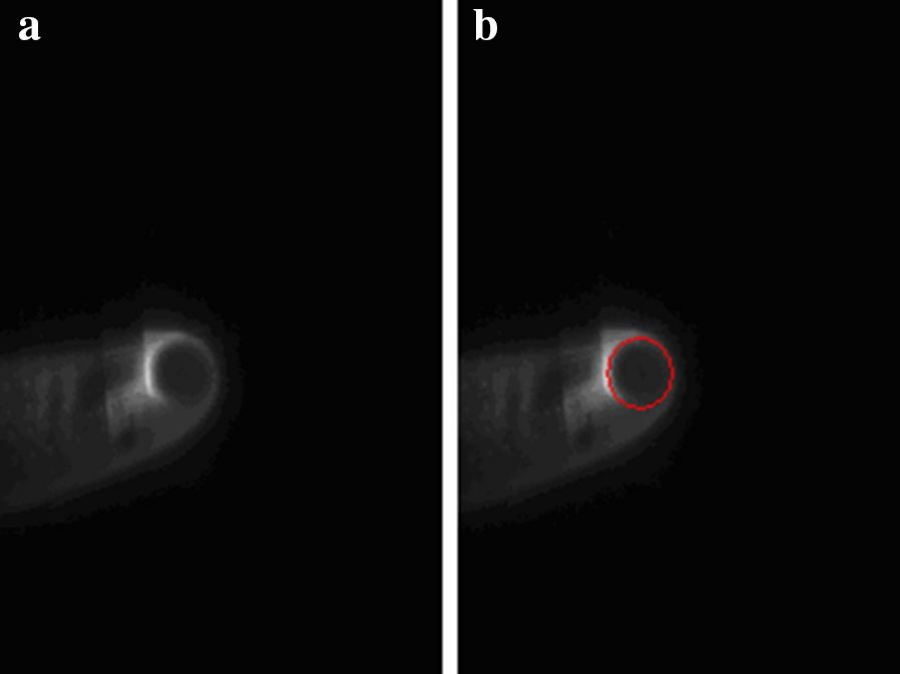
Image detection result at 0.58 s (EAST Shot#46965. **a** is the visible radiation image of plasma, and the red line in **b** is the image detection result)

**Fig. 9:**
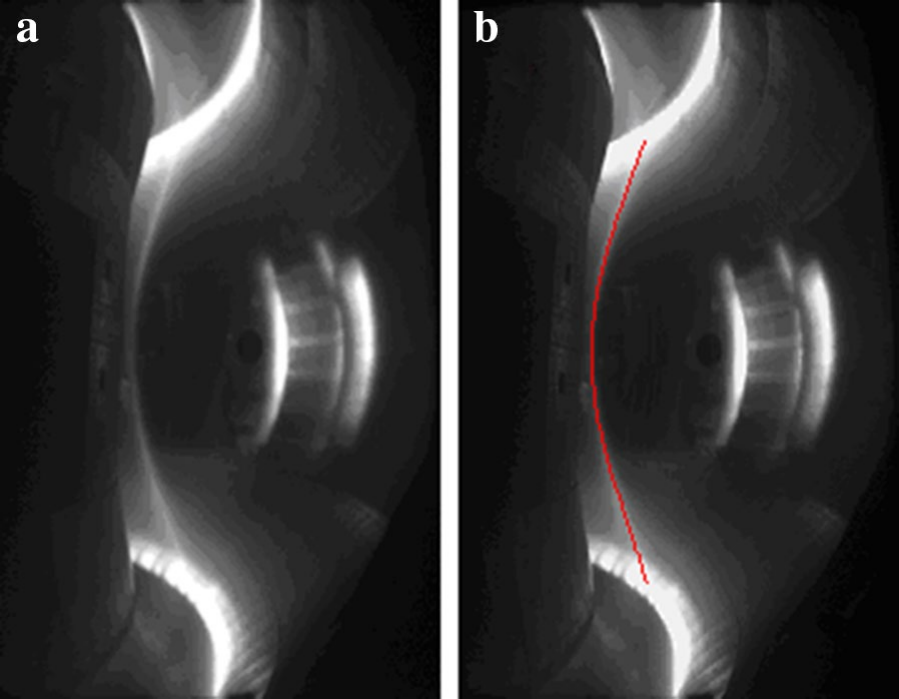
Image detection result at 4.43 s (EAST Shot#46965. **a** is the visible radiation image of plasma, and the result of boundary detection and curve fitting is shown in **b** with the red line)

**Fig. 10:**
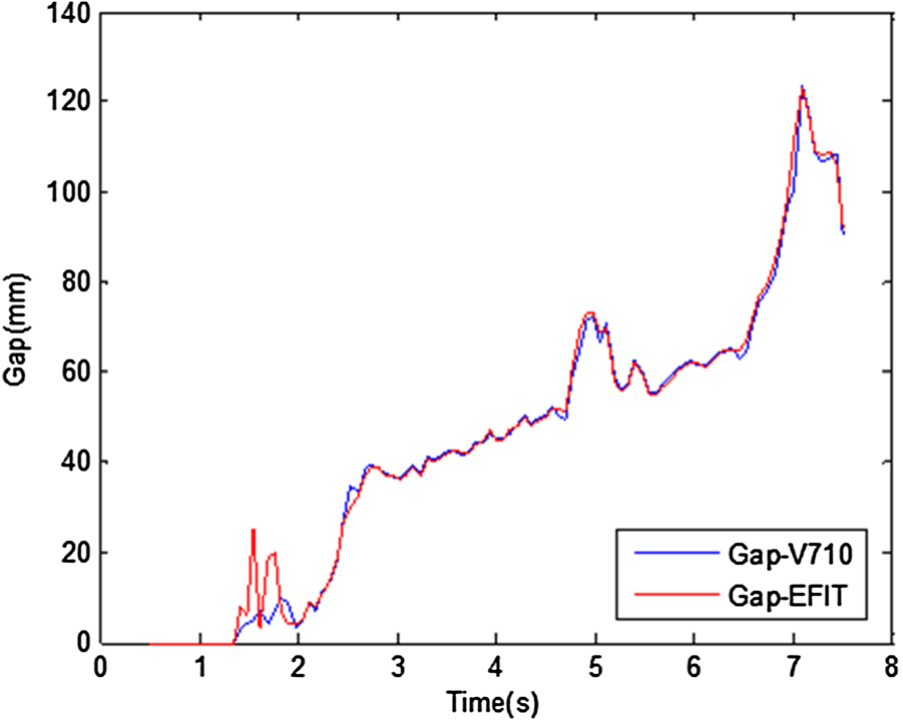
Time evolution of Gap on EAST shot# 46965 (Gap-EFIT is the result of Gap calculated by EFIT, and Gap-V710 is the result of Gap calculated by V710 images)

The EAST shot #46965 has been taken as an example. Figure 8a is the visible radiation image of plasma at time 0.58 s in discharge. Using above-mentioned algorithm, the plasma edge in red is shown in Fig. 8b. Combining with the spatial position of image, the Gap is about 14.40 mm. Figure 9a is the visible radiation image of plasma during the ascending discharge stage at time 4.43 s, and the Gap is about 31.68 mm. The result of boundary detection and curve fitting is shown in Fig. 9b. During the discharge process, the distance between the last closed flux surface (LCFS) and high field side inner wall of mid-plane in the device, or the Gap, is a very important physical parameter for plasma position control and the research on the interaction between the plasma and wall. Based on image detection and image calibration, the higher accuracy of Gap is given by using visible plasma image detection. In Fig. 10, the time evolution of Gap of EAST shot #46965 is given. In comparison with the Gap inversed by EFIT, the Gap obtained by the plasma image processing is very close to the inversed Gap by EFIT. Because of electromagnetic measurements interference, the two Gaps obtained by two methods are not equal. The Gap obtained through image detection is more precise and valuable for experiment analysis in the early stage of discharge.

The visible/IR cameras system has been put into use in EAST campaign. With the above-mentioned algorithm, the EAST discharge plasma image is identified and it can get excellent results. It mainly identifies circular plasma during the start-up state and the high field outer closed magnetic surface under configuration in divertor. Combining with the calibration of space position of the image, the time evolution of the distance (Gap) is also obtained. It can provide reference to plasma shape control. The improved plasma position control system has been designed and implemented using the fast CCD. The future control system should solve the Tokamak circuit equation using appropriate models to establish the horizontal and vertical magnetic fields, which will be sequentially compared to the optimal values. The control algorithm should also be studied to solve the current in the poloidal field coils by adjusting the magnitude and changing rate of the current of poloidal coils to ensure the steady-state operation of Tokamak plasma. In addition to the present hardware and software setups, a real-time computer is also needed for the acquisition of electromagnetic measurement data, and the control instructions should be sent to the poloidal field coils power system through real-time computing and data processing, fast enough to meet the demands of plasma control.

## Conclusions

The visible camera image acquisition and processing system is a practical system for real-time monitoring and for the controlling of the plasma discharge process, which is essential to the tokamak experiments. According to the structure of the EAST device and the position of the observation window, the fast camera image acquisition system was designed. The visible camera and infrared camera share the same window to observe the plasma discharge. The visible camera system is described in detail. As for the plasma edge detection, first pre-processing is performed to eliminate noise. Then the modified iteration threshold value is used to eliminate background and other useless information. After that, the improved Sobel algorithm is used to detect the configuration of plasma, and the plasma boundary location is obtained through the visible camera calibration, which can provide reliable data for feedback control during long-time plasma discharging. The experiment results during the EAST discharge show that the method of acquiring plasma position and boundary proposed in this paper has a high accuracy, which plays great importance on the EAST plasma position control.
